# Chitosan Nanoparticles Loaded with N-Acetyl Cysteine to Mitigate Ozone and Other Possible Oxidative Stresses in Durum Wheat

**DOI:** 10.3390/plants10040691

**Published:** 2021-04-02

**Authors:** Valentina Picchi, Serena Gobbi, Matteo Fattizzo, Mario Zefelippo, Franco Faoro

**Affiliations:** 1 CREA Research Centre for Engineering and Agro-Food Processing, Via G. Venezian 26, 20133 Milan, Italy; 2 Department of Agricultural and Environmental Sciences, University of Milano, Via Celoria 2, 20133 Milano, Italy; serena.gobbi@unimi.it (S.G.); matteo.fattizzo93@gmail.com (M.F.); 3 Department of Food, Environmental and Nutritional Sciences, University of Milano, Via Celoria 2, 20133 Milano, Italy; 4 Agronomist and Agricultural Consultant, Via S. Francesco D’Assisi 7/A, 27058 Voghera, Italy; mz@zefelippo.it

**Keywords:** chitosan, nanoparticles, NAC, oxidative stress, ozone, wheat

## Abstract

Modern durum wheat cultivars are more prone to ozone stress because of their high photosynthetic efficiency and leaf gas exchanges that cause a greater pollutant uptake. This, in turn, generates an increased reactive oxygen species (ROS) production that is a challenge to control by the antioxidant system of the plant, therefore affecting final yield, with a reduction up to 25%. With the aim of mitigating oxidative stress in wheat, we used chitosan nanoparticles (CHT-NPs) either unloaded or loaded with the antioxidant compound N-acetyl cysteine (NAC), on plants grown either in a greenhouse or in an open field. NAC-loaded NPs were prepared by adding 0.5 mg/mL NAC to the CHT solution before ionotropic gelation with tripolyphosphate (TTP). Greenhouse experiments evidenced that CHT-NPs and CHT-NPs-NAC were able to increase the level of the leaf antioxidant pool, particularly ascorbic acid (AsA) content. However, the results of field trials, while confirming the increase in the AsA level, at least in the first phenological stages, were less conclusive. The presence of NAC did not appear to significantly affect the leaf antioxidant pool, although the grain yield was slightly higher in NAC-treated parcels. Furthermore, both NAC-loaded and -unloaded CHT-NPs partially reduced the symptom severity and increased the weight of 1000 seeds, thus showing a moderate mitigation of ozone injury.

## 1. Introduction

Tropospheric ozone (O_3_) is a secondary air pollutant that is formed in reactions driven by the energy of solar radiation, involving the precursors nitrogen oxides (NOx), volatile organic compounds (VOCs, including methane), and carbon monoxide [1]. Although air pollutant emissions in Europe are likely to decline [2], the projected accumulated exposure over a threshold of 40 ppb (AOT40) for the period 2040–2059 has been estimated to exceed 3000 ppb-hours, particularly in the Mediterranean area [3].

O_3_ is the most important gaseous air pollutant and affects several crops, including wheat [4]. The global yield of wheat was reduced by up to 15% for the year 2000 due to O_3_ pollution [5], and in Italy the reduction has been much higher, often up to 25% [6]. O_3_ concentrations are projected to remain enhanced in many regions in the future, posing a threat to global food security [7]. significant variation in ozone sensitivity among wheat cultivars exist, and old cultivars not subjected to plant breeding appear less sensitive to the pollutant [8,9]. Furthermore, the higher photosynthetic efficiency and leaf gas exchanges of modern and most productive cultivars inevitably cause a greater pollutant uptake. This in turn generates an increased reactive oxygen species (ROS) production that is a challenge to control by the antioxidant system of the plant, therefore affecting the final yield. The differential response among cultivars also appears to be linked to the constitutive content of antioxidant metabolites, i.e., ascorbate [10], to the activity of enzymatic antioxidants [11,12], and/or to the capability of a cultivar to sense the stress at an early stage, with up-regulation of its antioxidant response [13]. When ROS production overwhelms antioxidant defenses, cells undergo severe damage, and, finally, cell death occurs. These damages may result in leaf symptoms on sensitive plants. Most wheat cultivars show typical leaf symptoms, characterized by small regular chlorotic spots that usually appear at mid-end of April and evolve in necrotic lesions, which are often mistaken for pathogen attack. However, thorough histo-cytochemical investigation excluded the presence of biotic factors as a cause of leaf lesions, confirming that the symptoms were likely due to an atmospheric pollutant [14]. Interestingly, in the presence of high UV radiation, symptoms could be worsened as a consequence of the synergistic oxidative stress which determines a higher and uncontrollable increase in ROS concentration [10].

Durum wheat (*Triticum durum*) is one of the most adapted cereals to the Mediterranean environmental conditions and its O_3_ sensitivity is often lower than that of common wheat [15]. Unfortunately, O_3_-tolerant (asymptomatic) cultivars are rarely cultivated because they are less productive. It is then highly desirable that geneticists, when selecting for new cultivars of durum wheat, consider not only their photosynthetic capacity but also their detoxification ability. Choosing crop cultivars with O_3_ tolerance or breeding new cultivars with this trait may be an important opportunity to reduce O_3_-induced agricultural losses [5]. This problem has not yet been addressed and genetic breeding requires a long development period. Nonetheless, agronomic approaches and methodologies to protect plants against O_3_ damage are urgently needed, given that an increase in production of even a few percent would have a significant effect at the global level. Among the tested chemicals, many pesticides have been used to protect plants against O_3_-induced phytotoxicity. However, low doses of stresses were found to stimulate not only plants, but also microorganisms, insects, and other potential agricultural pests, leading to pesticide tolerance or resistance in subsequent generations [16,17]. Several antioxidants have been also tested (e.g., ascorbic acid), in addition to physical barriers (e.g., antitranspirants and oils) and antiozonants (e.g., ethylenediurea) (reviewed by [18]). The potential application of nanomaterials represents a novel tool for protecting plants against O_3_-induced phytotoxicity. Nanoparticles (NPs) can be taken up passively through natural plant openings such as stomata, and then transported with apoplastic and/or simplistic movements to the xylem and phloem vessels, to reach tissues and organs [19]. Metallic NPs, such as gold and silver NPs, have been widely introduced in plant science for different applications, but their chemical synthesis is costly and requires the use of hazardous chemicals [20].

Chitosan (CHT) is a natural biopolymer, and a biodegradable and nontoxic compound. It is a deacetylated chitin derivative, which exerts its activity both as a plant resistance activator and fungitoxic compound. The former property is due to the induction of localized micro-oxidative bursts in treated plants [21], and fungal toxicity is possibly the consequence of an increased membrane permeability, together with the chelation of essential nutrients and the binding to DNA [22]. In the form of nanoparticles, the properties of CHT biopolymer can be further enhanced. Malerba and Cerana [23] reviewed the recent results on the application of CHT nanoparticles on plant productivity. CHT nanoparticles were effective in inducing the expression of pathogenesis-related proteins, thus enhancing the resistance against *Fusarium andiyazi* [24] and *F. oxysporum* [25]. In addition, the antifungal activity of CHT nanoparticles was also confirmed in other recent studies [26,27]. Chandra et al. [28] demonstrated the ability of CHT nanoparticles to boost significant plant innate immune responses and various defense-related enzymes in excised leaves of *Camellia sinensis* floated in different solutions of CHT-NPs.

The positive effects of the encapsulations of active ingredients in the chitosan domain have been reported and indicate a suitable strategy in the promotion of sustainable agricultural practices. Oliveira et al. [29] reported that chitosan nanoparticles encapsulated with NO donor (S-nitroso-mercaptosuccinic acid), could alleviate the salt stress in the maize plant, probably because of the slower release of NO by the nano-formulation and the higher protection of the NO donor from heat and decomposition [30]. In this study, we attempted to control ozone oxidative stress in durum wheat by treatment with CHT, CHT-NPs, and CHT-NPs loaded with the antioxidant N-acetyl cysteine (NAC), also with the aim to shed light on the mechanisms of NP activity. The results of the current research could have a greater impact, because in open fields other oxidative stress factors could act simultaneously, such as UV radiation, water stress, or the presence of other types of pollutants.

## 2. Results

### 2.1. Characterization of CHT-NPs and CHT-NPs-NAC

**Figure 1:**
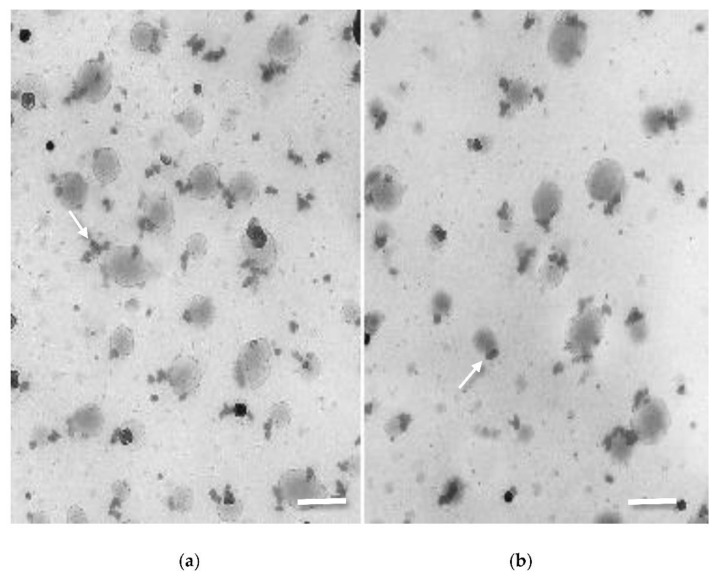
TEM images of (**a**) chitosan nanoparticles (CHT-NPs) and (**b**) CHT-NPs-N-acetyl cysteine (NAC) showing the presence of particles of 35–40 nm (arrows), aggregating to form larger particles of 150–200 nm (bars = 200 nm).

CHT-NPs prepared with the ionotropic gelation method were analyzed in terms of particle size and distribution using dynamic light scattering and transmission electron microscopy (TEM). CHT-NPs showed a mean diameter size of 167.5 ± 31.2, a polydispersity index (PDI) of 0.29, and a Z potential of 31.2 mV, whereas CHT-NPs-NAC were only slightly larger, with a mean size of 178 ± 28.6, a PDI of 0.397, and a Z potential of 47.2. Transmission electron microscopy carried out soon after preparation (Figure 1a) showed the presence of two different particle size populations: a fraction of small particles (around 30–40 nm) and a second population of 150–200 nm. The presence of two populations of particles was also shown by Rampino et al. [31], which demonstrated the aggregation of small particles into larger ones over time and after continuous stirring. No significant differences were observed in particle morphology after NAC loading (Figure 1b).

### 2.2. Effect of CHT Nanoparticles in Greenhouse Experiments

**Figure 2:**
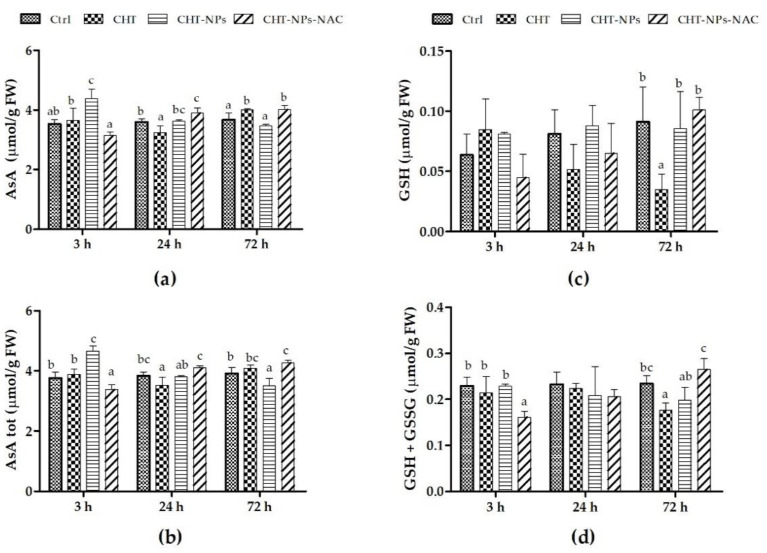
Contents of (**a**) reduced ascorbate (AsA), (**b**) total ascorbate (AsA + DHA), (**c**) reduced glutathione (GSH), and (**d**) total glutathione (GSH + oxidized glutathione (GSSG)) in three-week old seedlings of *Triticum durum* cv. Fabulis at 3, 24, and 72 h after treatments with water (Ctrl), CHT, CHT-NPs, and CHT-NPs-NAC. Vertical bars indicate SD (n = 3). At each time point, different letters indicate statistically significant differences among treatments, according to a one-way ANOVA followed by Least Significance Difference (LSD) post-hoc test (*p* < 0.05).

The results of reduced ascorbate (AsA) and total ascorbate (AsA + dehydroascorbic acid (DHA)) contents are shown in Figure 2a,b. CHT alone increased the AsA level compared to controls 3 h (+3.8%) and particularly 72 h (+9.1%) after the treatment. Leaves sprayed with CHT-NPs showed an increase in AsA and AsA + DHA (+24.5% and 23.5%, respectively) 3 h after treatment, but the differences were not statistically significant at 24 and 72 h compared to controls. In contrast, CHT-NPs-NAC induced a slight decrease in AsA and AsA + DHA 3 h after treatment, whereas at 24 and 72 h the level of AsA (and consequently of AsA + DHA) in CHT-NPs-NAC-treated leaves significantly increased compared to controls (+8%–9%). Reduced glutathione (GSH) and total glutathione (GSH + GSSG) pools did not significantly differ in CHT-NPs or CHT-NPs-NAC compared to controls (Figure 2c,d), except for a slight variation of oxidized glutathione (GSSG), which decreased at 3 h after the treatment with CHT-NPs-NAC. By comparison, the level of GSH was significantly reduced at 72 h in plants treated with CHT alone compared to controls. Based on these results, in the field experiment we decided to evaluate the effect of CHT-NP and CHT-NP-NAC 48 h after the treatment.

### 2.3. Effect of CHT Nanoparticles in Field Experiments

#### 2.3.1. Pollutant and Climate Monitoring

**Figure 3:**
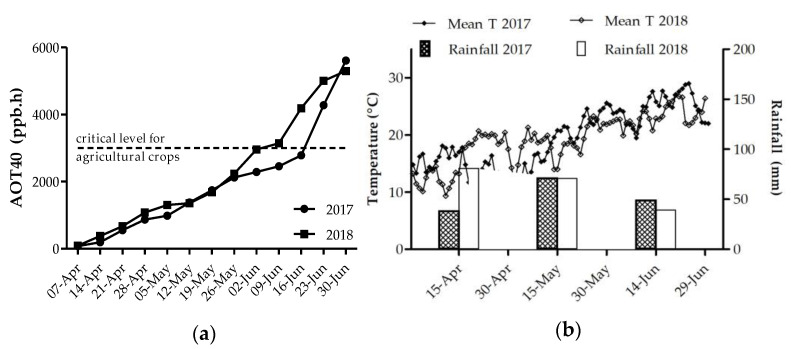
(**a**) Accumulated dose over a threshold of 40 ppb (AOT40) of ozone and threshold of 3000 ppb.h (critical level for crop species not to be exceeded in three months); (**b**) rainfall distribution during the two years of the survey (April–June, each year) and mean daily temperature (Mean T).

AOT40 values from the beginning of April until the end of June were similar in both 2017 and 2018 (5296 and 5605 ppb.h, respectively) (Figure 3a). However, although in 2018 the critical level of 3000 ppb.h for agricultural crops was exceeded on the 17th of June, in 2017 the same level was reached ten days earlier, the 7th of June. Monthly rainfall and mean daily temperature are shown in Figure 3b. Compared to 2017, in 2018 rainfall was heavier in April, and in May and June total rainfall was very similar. In the first half of April, temperatures were lower in 2018, whereas from the second half of April until mid-May, 2018 was warmer compared to 2017. In contrast, starting from mid-May until harvest, the mean daily temperatures of 2018 were lower compared to 2017.

#### 2.3.2. Leaf Visible Injuries

**Figure 4:**
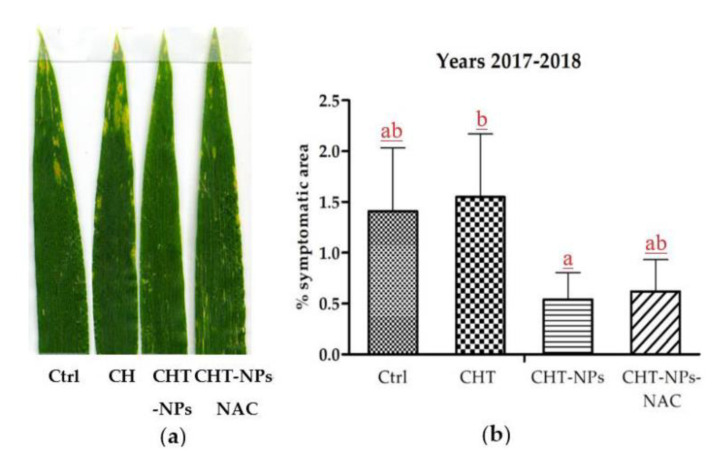
(**a**) Representative samples of leaves from different treatments and showing different degrees of chlorotic or necrotic lesions typical of ozone injury. (**b**) Results of image analysis of symptomatic leaves a week after the third treatment; data of 2017 and 2018 treatments have been pooled for statistical analysis. Different letters indicate statistically significant differences among treatments, according to a one-way ANOVA followed by LSD post-hoc test (*p* < 0.05).

The first visible symptoms appeared on the leaf surface during the first 10 days of May in both 2017 and 2018, although in a mild form, as the AOT40 was still below the critical level of 3000 ppb.h. Afterwards, they increased in intensity, particularly in control and CHT parcels, and were milder in plants treated with either CHT-NPs and CHT-NPs-NAC. However, after the third treatment, CHT-NPs appeared to be more effective than CHT-NPs-NAC in reducing the percentage of symptomatic leaf surface, as shown in Figure 4a.

The image analysis of symptomatic leaves was carried out a week after the third treatment in both years, and data of 2017 and 2018 were pooled for statistical analysis, as shown in Figure 4b. Due to the high variance within leaves, significant differences were found only between CHT and CHT-NPs treatments, although a tendency toward symptom reduction was observed also in CHT-NPs-NAC. Instead, CHT treatment showed a tendency to increase symptom severity in respect to control plants treated with water.

#### 2.3.3. Leaf Antioxidant Content

**Figure 5:**
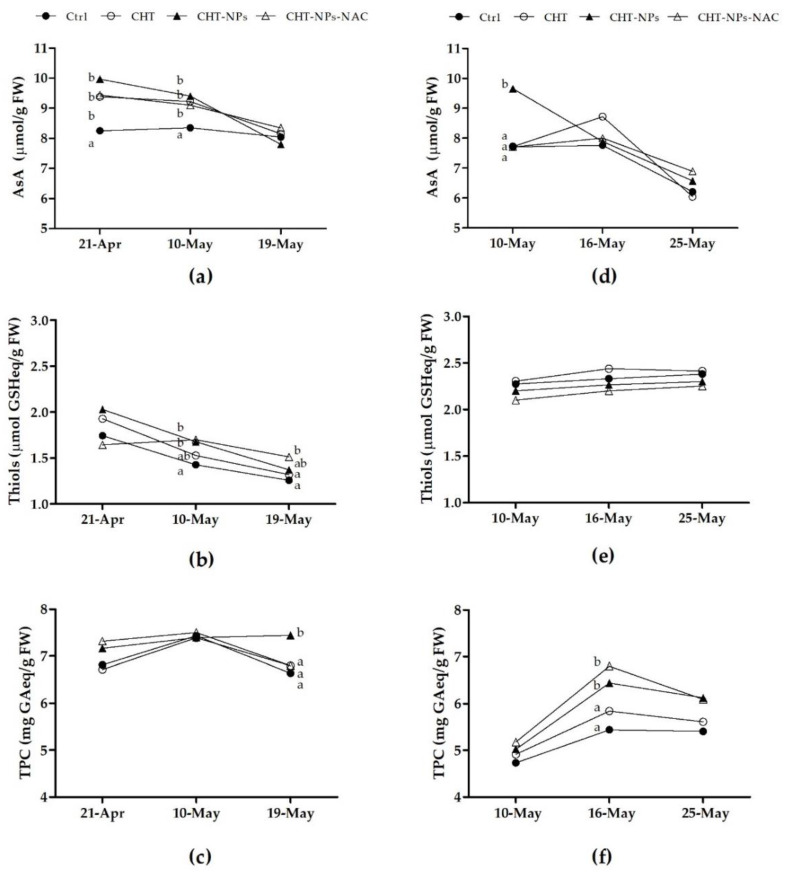
Contents of reduced ascorbate (AsA), thiols, and total polyphenols (TPC) in *Triticum durum* cv. Fabulis untreated (controls) or treated with CHT, CHT-NPs, and CHT-NPs-NAC, at the three sampling dates of 2017 (**a**–**c**) and 2018 (**d**–**f**). At each time point, different letters indicate statistically significant differences among treatments, according to a one-way ANOVA followed by LSD post-hoc test (*p* < 0.05).

The results of AsA, total thiols, and total polyphenols measured in 2017 are shown in Figure 5a–c).

At first (21th April) and second (10th May) sampling dates, AsA content significantly increased in plants treated with CHT, CHT-NPs, and CHT-NPs-NAC compared to controls (+10%–20%, on average) (Figure 5a). The plants treated with CHT-NPs and CHT-NPs-NAC on the 10th May had higher total thiols compared to controls (Figure 5b), and 48 h after the third treatment (19th May), plants treated with CHT-NPs-NAC still showed a significantly higher total thiol content (+20.3%) compared to controls. During the second year of experiment, the effect of NP application on leaf antioxidants was less clear, but treatment with CHT-NPs confirmed its positive effect in increasing the AsA level of flag leaves, at least at the first sampling date (Figure 5d). Regarding total polyphenols, in 2017 we observed a significant increase following CHT-NP treatment at the third sampling date (Figure 5c), whereas in 2018 total polyphenols were higher in plants treated with CHT-NPs and CHT-NPs-NAC at the second sampling time (Figure 5f).

#### 2.3.4. Crop Yield

Table 1 reports the values of the crop yield parameters measured in *Triticum durum* cv. Fabulis at the end of each field experiment. One-way ANOVA shows that the treatment did not significantly influence the grain yield or the hectoliter weight in 2017 and 2018, whereas the 1000-grain weight was positively influenced by the treatments in both years. In particular, plants treated with CHT-NPs showed a significant increase in this parameter (+5.3% and +6.8%, in 2017 and 2018, respectively), in addition to plants treated with CHT-NPs-NAC (+3.8% and +6.6%, in 2017 and 2018, respectively).

## 3. Discussion

On a global scale, O_3_-induced relative yield loss for wheat (i.e., yield loss compared to a theoretical yield without O_3_ damage) ranges from 3.9% to 15%, with peaks of 17% for South Asia [5]. Breeding new O_3_-tolerant cultivars may provide an important opportunity to reduce O_3_-induced agricultural losses. To date, however, the problem has not been specifically addressed. For this reason, agricultural practices that may mitigate oxidative stress and make the plants more tolerant to O_3_ would be helpful, particularly if they are low-cost and easy to implement.

Chitosan, a natural polymer, has been recognized as an effective biotic elicitor that induces the systemic resistance in plants [22]. To date, chitosan in the form of nanoparticles has not been examined for its plant elicitor activity as much as in its natural form. Recently, Kheiri et al. [32] compared the efficacy of CHT and CHT-NPs in controlling Fusarium head blight (FHB) disease caused by *Fusarium graminearum* in wheat. Interestingly, they found that at the same concentration, CHT-NPs better inhibited fungal growth than CHT. In our work we showed that chitosan in the form of nanoparticles can also activate effective plant defense responses against oxidative stress in the open field and can accomplish a better efficacy compared to natural chitosan. The method for the preparation of CHT-NPs and CHT-NPs-NAC allowed the production of homogeneous and almost spherical NPs, characterized by a low polydispersity index, with dimensions (range 30–200 nm) small enough to enter through the stomatal pores and possibly unload their content into mesophyll intercellular spaces.

In the first part of the study, we tested NPs in a greenhouse experiment on young wheat seedlings, with the aim of assessing the potential of the treatments to induce changes in the leaf antioxidant pool. Plants were then sprayed with CHT alone, CHT-NPs, and CHT-NPs-NAC. Our results showed that CHT alone increased ascorbate content 72 h after the treatment, whereas CHT-NPs exerted a positive effect on ascorbate content at 3 h. CHT-NPs-NAC enhanced the ascorbate level 24 h after the treatment, and this positive effect was maintained 72 h after the spray. CHT is known to trigger the production of hydrogen peroxide [33], which acts as a messenger for the transcription of defense-related genes. The enhancement of ascorbate level, as observed in the greenhouse experiment, can be a consequence of the plant attempting to control the H_2_O_2_ level through the increased activity of APX, the enzyme that catalyzes the conversion of H_2_O_2_ to water by using ascorbate as an electron donor. The application of CHT in the form of NPs resulted in a faster increase in ascorbate level compared to CHT alone, thus suggesting that NPs were more efficient in inducing an early response of the plants compared to natural CHT. Moreover, the results of CHT-NPs loaded with NAC evidenced a positive effect in maintaining a higher ascorbate level for longer times, i.e., until 72 h after spraying. Our preliminary results suggest a possible positive effect of CHT-NP application in improving plant resistance to oxidative stress, because higher ascorbate content in durum wheat varieties have been related to a greater tolerance to O_3_ exposure [10,12].

Compared to AsA, GSH and GSSG were less influenced by CHT or CHT-NPs. The greenhouse experiment indicated that GSH biosynthesis was not directly stimulated by the application of CHT-NPs. However, the tendency towards an increase in the GSH + GSSG pool was observed 72 h after the treatment with CHT-NPs-NAC. This result may be due to the incremented cysteine availability induced by NAC application, and suggests that CHT-NPs-NAC treatment may provide protection to plants against oxidative stress. GSH, in fact, is an antioxidant metabolite of great importance, because it directly controls ROS production, and also indirectly reduces the power of glutathione peroxidase (GPX) and recycles the ascorbate pool, through the sequence of reactions collectively known as the Halliwell–Asada or ascorbate–glutathione cycle.

Based on these promising results, we analyzed the potential positive effect of CHT-NP treatments on an O_3_-sensitive wheat variety, grown in an open field in the Po valley (Northern Italy). In Southern European regions, the critical level for agricultural crops is often exceeded at the end of April, when wheat is in the phenological phase of anthesis and the stomatal conductance reaches its maximum value [34,35]. The field experiment was conducted in the period April–June in 2017 and 2018. In both years, and particularly in 2018, we registered a relatively low O_3_ level during anthesis, and the critical level for agricultural crops of 3000 ppb.h was exceeded in the first week of June, i.e., at complete maturity for plants. The relatively low O_3_ concentrations caused the appearance of only mild symptoms on wheat leaves. In a previous study, two sensitive wheat cultivars, Artico (common wheat) and Virgilio (durum wheat), which were grown in the same geographic area of the Po Valley, showed symptoms covering over 20%–25% of the leaf area [14]. Nevertheless, CHT-NP and CHT-NPs-NAC parcels showed on average a lower symptomatic leaf area compared to controls, thus showing a moderate mitigation of ozone injury. After the third treatment, CHT-NPs were found more effective than CHT-NPs-NAC in reducing the symptomatic leaf surface. We can hypothesize that the presence of NAC can partly overwhelm the capacity of CHT to induce stomatal closure [36], possibly neutralizing the CHT-induced micro-oxidative bursts responsible for the activation of plant defense.

Regarding the leaf antioxidant pool, we observed that all treatments, particularly CHT-NPs, confirmed their positive effect in increasing AsA content. The effect was more pronounced in the first year of experiment and particularly in the period before anthesis. By comparison, total thiols were more influenced by CHT-NPs-NAC treatment, probably because of the higher cysteine availability, due to the NAC presence. However, the effect on total thiols was observed only in the first year of experiment. Because the AOT40 level between April and the end of May was very similar in 2017 and 2018, the higher induction of an antioxidant response observed in 2017 could be due to the different weather conditions and, in particularly, to the generally lower temperatures of April and May. In fact, it appears that the impact of O_3_ is greater with colder temperatures. Hansen et al. [37], for example, revealed a strong dependence of wheat phenological development on temperature, and reported that plants grown in 5-degree colder treatments were more affected by O_3_ compared to plants grown in 5-degree warmer treatments. In addition, higher temperatures and higher insolation, i.e., factors that influence the stomatal conductance, can alter the flux of O_3_ into mesophyll, thus decreasing the relative impact of the pollutant [38]. For these reasons, in 2017 the weather conditions may have determined a general lower oxidative stress in plants, so the physiological responses of wheat plants were not triggered.

CHT is known to elicit plant resistance mechanisms through a long lasting and systemic immunity (systemic acquired resistance, SAR), which includes the synthesis of secondary metabolites such as phenolic compounds (other than callose), phytoalexins, and pathogenesis-related (PR) proteins. It also includes the modulation of the activity of several enzymes involved in detoxification processes and plant defense barriers (phenylalanine ammonia-lyase (PAL), chitinase, polyphenol-oxidases (PPOs) and peroxidases such as guaiacol-peroxidase (G-POD) and ascorbate peroxidase (APX)) [39]. In our study, despite some different trends observed in the two years, the results evidenced an activation of the phenylpropanoid pathway following CHT-NP and CHT-NPs-NAC applications, but not after CHT treatment. In particular, the total phenolic content was increased following CHT-NPs in both years, whereas CHT-NPs-NAC seemed to exert a positive influence on phenolics only in the second year. Similar results were observed in the study by Chandra et al. [28], in which the induction of total phenol content in CHT-NP-treated *Camellia sinensis* leaves was found to be 3.5% higher than that of chitosan-treated leaves. The same authors also observed an increase in the accumulation of gallic acid (GA), epicatechin (EC), epigallocatechin (EGC), and epigallocatechin gallate (EGCG) in the treated leaves compared to those of the untreated controls.

Regarding the effect of the treatments on the final wheat yield, a slight tendency towards an increase was observed in the first year of the field experiment in parcels treated with CHT-NPs-NAC, but the results of the present study did not evidence a clear positive effect of NPs on crop yield. The potential of chitosan oligosaccharides (COS) in protecting wheat yield from the negative impact of abiotic stress was previously studied by Wang et al. [40]. The authors showed that COS can impact wheat production in the field by improving the yield components, and that tillering and returning-green stage were the most sensitive to COS spraying. In fact, when COS were applied at tillering and returning-green stages, they significantly improved the grain yield, through an increase in spike number and grains per spike. In the present study, chitosan was applied at later phenological phases, i.e., when the flag leaf was already present, and this different timing could explain the different findings. In contrast to our results, Wang et al. [40] did not observe a positive impact of chitosan foliar application on 1000-grain weight. Our results, instead, showed that both CHT-NP and CHT-NPs-NAC applications induced a significant increase in the 1000-grain weight, evidencing a positive impact on the wheat grain quality, because this parameter is related to dry matter accumulation and partitioning.

## 4. Materials and Methods

### 4.1. Production of Chitosan Nanoparticles (CHT-NPs and CHT-NPs-NAC)

CHT-NPs were prepared through the ionotropic gelation as described in Rampino et al. [31] with some modification, using pentasodium tripolyphosphate (TPP, Acros Organics) as ion-cross linker. Briefly, low molecular weight chitosan (161 kDa, 90% N-deacetylation, Bio Basic Inc., Markham, ON, Canada) was dissolved in 0.05% *v/v* acetic acid at concentration 0.5 mg/mL and adjusted to pH 5.6 using NaOH. Aqueous solution of TPP (0.5 mg/mL) was added dropwise to the CHT solution under constant stirring, up to a CHT:TPP molar ratio of 3:1. CHT nanoparticles (NPs) formation was evidenced by the appearance of an opalescent solution. CHT-NPs loaded with NAC were prepared as above but NAC (0.2 mg/mL) was added to the CHT solution before ionotropic gelation. Nanoparticle suspensions were then centrifuged at 10,000 rpm for 30 min and resuspended in distilled water adjusted to pH 5.6 with acetic acid to the same final volume of chitosan solution used for their preparation.

### 4.2. Nanoparticle Characterization

#### 4.2.1. Morphology

Chitosan nanoparticle suspensions were deposited on 300 mesh nickel grids precoated with carbon and collodion films, which were then allowed to dry. Afterwards, grids were stained with 2% Uranyl acetate and examined with a Jeol 100SX transmission electron microscope (Jeol, Japan).

#### 4.2.2. Particle Mean Size Determination

Mean particle size, polydispersity index (PDI), and zeta potential were assessed by a Nanotrac Wave II (Microtrac MRB, Haan, Germany). The analysis was performed at 25 °C, and each nanoparticle dispersion was measured in triplicate and reported as the mean ± standard deviation.

#### 4.2.3. HPLC Measurements of NAC Concentration

The content of NAC in CHT-NAC-NPs was analyzed by HPLC with coulometric electrochemical detection (ESA mod. 6210, Chelmsford, MA, USA), following the protocol by Yap et al. [41], with slight modifications. The isocratic elution was carried out using 25 mM monobasic sodium phosphate containing 0.5 mM heptan-sulfonic acid (ion-pairing agent) and 0.25% acetonitrile. The value of pH was adjusted to 2.7 with 85% phosphoric acid. An aliquot of CHT-NPs-NAC was properly diluted and 20 µL of filtered solution was injected to HPLC. A flow rate of 0.6 mL/min was used with a C18 column (5 µM column, 4.6 × 250 mm). The four-array electrode system was 1 = +300, 2 = +450, 3 = +600, 4 = +900 mV. Electrodes 1 and 2 served as screening electrodes to oxidize potentially interfering compounds. NAC was detected on electrodes 3 and 4. Quantification was made using a known concentration of NAC solution (range 0.005–0.05 mg/mL).

### 4.3. Greenhouse Experiments

#### 4.3.1. Plant Material and Treatments

Seeds of durum wheat cv. Fabulis were sown in pots filled with perlite (two plants per pot). The plants were grown in an experimental greenhouse under monitored conditions (25 ± 3 °C, 14 h photoperiod). Three-week old seedlings, at the stage of 3–4 fully expanded leaves, were sprayed with four different treatments: (i) control (water), (ii) CHT (0.5 mg/mL in 0.05% acetic acid), (iii) CHT-NPs, and (iv) CHT-NPs-NAC. Treatments were prepared as described above but 0.01% Twin was added as a surfactant. The solutions were sprayed on the plants in order to completely cover the leaves. Leaf samples were collected at 3, 24, and 72 h after the treatments. Three replicates per treatment were collected each time. Samples were weighed, immediately frozen in liquid nitrogen, and analyzed.

#### 4.3.2. Ascorbic Acid and Glutathione Determination

Frozen foliar tissue (200 mg) was ground with liquid nitrogen in a pre-cooled pestle. The powder was added to 3 mL of 6% metaphosphoric acid (MPA). The homogenate was vortexed for 30 s, centrifuged at 12,000 rpm for 15 min at 4 °C, and filtered on a 0.45 µm filter. Extracts were then used for ascorbic and glutathione determination. L-ascorbic acid (AsA) was quantified by HPLC, as previously described [42]. The oxidized form (dehydroascorbic acid, DHA) was determined by the “subtractive” method after measurement of the total ascorbate content (AsA + DHA) following reduction with tris(2-carbossietil)fosfina (TCEP) 100 mM in HCl 1 N [43]. The isocratic elution was performed using 0.02 M orthophosphoric acid at a flow rate of 0.7 mL/min. Samples of 20 µl were injected and monitored at 254 nm. The ascorbic acid content was quantified by comparison with a standard curve obtained with known ascorbic acid concentrations. Reduced glutathione (GSH) and oxidized glutathione (GSSG) were detected using HPLC with a coulometric electrochemical detector following the same protocol as described in Section 4.2.3. GSH was detected on electrodes 3 and 4, and GSSG was monitored on electrode 4. Quantification was performed using a calibration curve of a standard mixture containing GSH and GSSG.

### 4.4. Field Experiments

#### 4.4.1. Site Description, Treatments, and Sample Collection

Durum wheat cv. Fabulis was grown over two consecutive seasons (2016–17 and 2017–18) in an experimental field situated near the city of Voghera, in North Italy (44°59′43″ N; 9°2′56″ E). Seeds were sown in December and plants reached the flowering stage in mid-May. The field was divided into 12 plots (10 m^2^ each) and each of the four treatments were randomized in triplicate. The solutions, previously added with Twin (0.01%), were sprinkled with a sprayer pump on the plants (about 1 L solution/plot). The treatments were: control (deionized water), chitosan 0.05 mg/mL, CHT-NPs, and CHT-NPs-NAC. Treatments were carried out three times at different phenological phases of the plants: at mid-April when flag leaves were not completely expanded (BBCH37), the first week of May at inflorescence emergence (BBCH53), and the second 10-day period of May during anthesis (BBCH65). Leaf samples (three replicates of ten leaves per treatment) were randomly collected 48 h after the treatments, immediately frozen in liquid nitrogen, and stored at −80 °C until analysis. Three replicates per plot were collected each time. Final harvesting of the crop was carried out at the end of June. For each plot, total grain yield and hectoliter weight were measured. Finally, the 1000-grain weight was assessed on three replicates per plot.

#### 4.4.2. Pollutant and Climate Monitoring

Continuous hourly measurements of ambient concentration of O_3_, temperature and rainfall were made by a nearby weather station equipped with a photometric O_3_ analyzer. Ozone exposure was expressed as AOT40 (accumulated dose over a threshold of 40 ppb, following De Leeuw and Zantvoort [44]).

#### 4.4.3. Analysis of Visible Symptoms

Leaf damage was assessed on flag leaves a week after the third treatments. The extent of foliar injury was assessed by estimating the injured adaxial leaf area on digitalized images at 300 dpi of ten randomly selected leaves per plot, with an image analyzer (Global Lab©, Data Translation, New York, NY, USA).

#### 4.4.4. Analysis of Ascorbic Acid and Total Thiols

Ascorbic acid (AsA) was determined as described in Section 4.3.2. The total content of free thiols (−SH groups) was measured following the method described by [45] with minor modifications. Extracts in 6% MPA (500 µL) were mixed with 1 mL of a Na/P buffer (1 M, pH 8) and 200 µL 10 mM DTNB (5,5′-dithiobis(2-nitrobenzoic acid)). The absorbance at 412 nm was immediately measured. Quantification was performed using a calibration curve of a standard solution of GSH and results were expressed as µmol GSH equivalent (GSHeq) per g of fresh weight.

#### 4.4.5. Analysis of Total Phenols

Total phenol content was determined using the Folin–Ciocalteu (FC) method as reported by [46]. Extracts were prepared according to [47]. Five mL of 1 M EtOH/HCl were added to 200 mg of ground frozen sample and vortexed for 1 min. Then the samples were heated at 80 °C for 3 h, vortexing every 30 min. After cooling, the samples were centrifuged for 5 min at 5000 rpm and filtered (0.45 µm filter). A quantity of 200 µL of extract was mixed with 4 mL of distilled water, 0.5 mL of FC reagent, and 1.5 mL Na_2_CO_3_ (7.5% *w*/*v*). Samples were allowed to stand in the dark at room temperature for 2 h and then the absorbance was measured at 730 nm, using an UV-Vis spectrophotometer (UVIDEC-320, Jasco, JP, Tokyo, Japan). The total phenolic content was expressed as mg of gallic acid equivalent (GAE) per g of fresh weight.

### 4.5. Statistical Analysis

The results of the biochemical analyses were expressed as mean ± standard deviation. Data were subjected to analysis of variance (ANOVA), and comparison among means was determined according to Least Significance Different (LSD) test. Significant differences were accepted at *p* < 0.05 and indicated with different letters. Statistical analysis was performed using the Statgraphics v.7 (Manugistic Inc., Rockville, MD, USA) software package.

## 5. Conclusions

The current study shows that CHT-NPs and, in particular, CHT-NPs loaded with NAC, were effective in increasing the ascorbate content in young wheat seedlings grown in a greenhouse, whereas in an open field the positive effect of CHT-NPs on leaf antioxidant pool appeared less dependent on the addition of NAC. We can hypothesize that the concentration of NAC of the field experiment, although the same as that used in greenhouse trials, was too low to induce a positive effect in mature plants. Nevertheless, the potential of chitosan nanoparticles to increase the leaf antioxidant pool both in greenhouse and open-field plants was demonstrated, probably because of the slower release of CHT due to the nano-formulation.

Further study is needed to better understand the mechanisms of CHT-NP and CHT-NPs-NAC bioactivity, in addition to their optimum concentration and time, to improve their effectiveness in the open field. Nonetheless, these two treatments have the potential to trigger plant defenses and protect plants from O_3_ stress, and other possible oxidative stresses, with a positive effect on the final yield.

## Figures and Tables

**Table 1:** Mean values ± standard deviation of crop yield parameters measured in 2017 and 2018 in *Triticum durum* cv. Fabulis untreated (Ctrl) or treated with CHT, CHT-NPs, or CHT-NPs-NAC.

		Grain Yield (t ha^−1^)	Hectoliter Weight (kg hl^−1^)	1000-Grain Weight (g)
2017	Ctrl	7.6 ± 0.1	79.6 ± 0.4	52.5 ± 1.8 a ^1^
	CHT	7.6 ± 0.5	79.1 ± 0.8	51.9 ± 2.0 a
	CHT-NPs	7.4 ± 0.7	80.2 ± 0.7	55.3 ± 1.3 b
	CHT-NPs-NAC	8.5 ± 0.4	79.8 ± 0.6	54.5 ± 1.4 b
2018	Ctrl	6.3 ± 0.5	77.8 ± 0.9	48.6 ± 1.4 a
	CHT	6.1 ± 0.5	76.5 ± 0.8	51.0 ± 1.0 b
	CHT-NPs	6.0 ± 0.4	77.3 ± 0.5	51.9 ± 1.1 b
	CHT-NPs-NAC	6.3 ± 0.4	77.7 ± 0.6	51.8 ± 1.1 b

^1^ For each year, means followed by different letters in the same column are significantly different according to LSD test (*p* < 0.05).
